# DeWitt County Medical Society

**Published:** 1869-06

**Authors:** John A. Edmiston

**Affiliations:** Secretary


### DeWITT COUNTY MEDICAL SOCIETY.

The Society met in annual session at the office of Drs. Goodbrake & Edmiston, in the City of Clinton.

The President, Dr. J. A. Edmiston, in the chair.

Minutes of last meeting read and approved.

Dr. W. G. Cochran, of Farmer City, was proposed for membership and elected.

Officers for the ensuing year:—President, Dr. John Wright; Vice-President, Dr. J. W. Edmiston; Treasurer, Dr. C. Goodbrake; Secretary, Dr. Edmiston; Censors, Drs. W. G. Cochran, C. Goodbrake, J. H. Tyler.

Dr. Wright, in a neat speech, returned thanks for the honor conferred upon him.

The retiring President, Dr. J. A. Edmiston, asked until next regular meeting to complete his annual address, which was, on motion, granted.

Drs. C. Goodbrake and John Wright were elected delegates to the American Medical Association for the next year; and Drs. C. Goodbrake, J. Wright, and B. S. Lewis were elected delegates to the Illinois Medical Society, with power to designate alternates in case of inability to attend.

Various subjects were brought before the meeting and discussed at length.

Drs. W. G. Cochran and J. H. Tyler were appointed essayists.

On motion, the Secretary was instructed to furnish copies of proceedings to the Clinton papers and Chicago medical journals, for publication.

On motion, adjourned until next regular meeting.

JOHN A. EDMISTON, *Secretary.*

